# KIF23 Overexpression Promotes Cell Viability, Migration, and Invasion via the Wnt/β‐Catenin Signaling Pathway in Anaplastic Thyroid Carcinoma

**DOI:** 10.1155/ije/7664607

**Published:** 2026-02-10

**Authors:** Yongkang Wu, Changwei Zheng, Weijie Chen, Tuo Xu

**Affiliations:** ^1^ Department of Vascular and Thyroid Surgery, Affiliated Hospital of Guangdong Medical University, Zhanjiang, Guangdong, China, gdmuah.com

**Keywords:** anaplastic thyroid carcinoma, ferroptosis, KIF23, oxidative stress, Wnt/β-catenin signaling pathway

## Abstract

**Background:**

Anaplastic thyroid carcinoma (ATC) is a rare, aggressive cancer with a poor prognosis and limited treatment options. KIF23, a key regulator of cell division, has been implicated in tumor progression, but its role in ATC remains unclear. This study investigates the effects of KIF23 on ATC cell viability, migration, invasion, and signaling pathways.

**Methods:**

ATC cell models were generated by transfecting cells with KIF23 silencing or overexpression plasmids. KIF23 expression was measured by RT‐qPCR and Western blot. Cell viability, oxidative stress, migration, and invasion were assessed using CCK‐8, ELISA, scratch, and Transwell assays. Wnt/β‐catenin pathway activation was analyzed by Western blot, and ferroptosis was induced by erastin.

**Results:**

Silencing KIF23 significantly decreased cell viability, increased oxidative stress, and reduced cell migration and invasion. Overexpression of KIF23 enhanced cell viability, migration, and invasion, and these effects were partially reversed by the Wnt/β‐catenin pathway inhibitor NTZ. KIF23 overexpression led to a decrease in intracellular ROS levels and reduced oxidative stress markers. Erastin treatment, in contrast, increased ROS levels, reduced cell viability, and suppressed migration and invasion. In the OE‐KIF23 + erastin group, erastin partially reversed the pro‐survival and pro‐motility effects of KIF23 overexpression, with ROS levels and functional readouts shifting toward those of erastin‐treated cells, indicating that KIF23 interacts with ferroptosis‐related oxidative stress regulation in ATC cells.

**Conclusion:**

KIF23 regulates ATC cell viability, migration, and invasion via the Wnt/β‐catenin signaling pathway and ferroptosis. These findings suggest that KIF23 may be a potential therapeutic target for ATC treatment.

## 1. Introduction

Anaplastic thyroid carcinoma (ATC) is a rare but aggressive endocrine malignancy that poses major challenges in clinical management [1]. It is characteristic by the rapid invasion, early metastasis, and poor prognosis, with a median survival time of only 4 months, a 6‐month survival rate of 35%, and a disease‐specific mortality rate nearing 100% [2, 3]. The pathogenesis of ATC remains incompletely understood, but multiple genetic and molecular alterations have been implicated, including TERT promoter and TP53 mutations, as well as frequent abnormalities in the PIK3/PTEN/AKT/mTOR pathway, SWI‐SNF complex, histone methyltransferases, and mismatch repair genes [4]. These abnormalities collectively contribute to the rapid proliferation, invasion, and metastasis of cancer cells. Despite the use of traditional modalities such as surgery, radiotherapy, and chemotherapy, therapeutic efficacy remains restrained due to the resistance to anticancer drugs and the rapid progression of ATC [5, 6]. Recently, targeted therapies have changed the landscape of ATC management. The combination of dabrafenib and trametinib has been approved for patients with BRAFV600E‐mutant ATC and has demonstrated rapid and significant tumor regression [3]. However, resistance to BRAF/MEK inhibitors inevitably develops. More recent consensus statements further highlight the importance of molecular profiling, the potential benefit of adding immunotherapy, and neoadjuvant approaches, but many of these advances have not yet been incorporated into standard guidelines [7]. Therefore, further exploration of the pathogenic mechanisms of ATC and the identification of novel therapeutic targets are crucial for improving patient outcomes and enhancing treatment effectiveness.

Advancements in molecular biology and sequencing technology have greatly deepened our understanding of gene variations and abnormal expressions in tumor cells, providing novel therapeutic strategies. Kinesin family Member 23 (KIF23), also known as mitotic kinesin‐like Protein 1 (MKLP1), is a crucial kinesin family member that drives intracellular transport and cell division through ATP hydrolysis along microtubules [8, 9]. Increasing evidence indicates that KIF23 plays an oncogenic role in multiple malignancies. Studies have indicated that KIF23 expression was markedly increased in several tumor types, including endometrial cancer and triple‐negative breast cancer, which promotes proliferation, migration, and epithelial–mesenchymal transition (EMT) and correlates with poor prognosis [10–12]. Besides abnormal expression levels, genetic mutations in KIF23 have also been linked to tumorigenesis. These mutations may alter the protein structure and function of KIF23, thereby affecting the normal progression of cell division and providing conditions for tumor formation [13, 14]. Owing to these roles, KIF23 has attracted attention as a potential therapeutic target. Some studies explore ways to inhibit tumor cell proliferation and spread by targeting KIF23 function [15, 16]. In ATC specifically, a study has suggested that SIRT7 promoted the proliferation and migration of ATC cells by regulating the desuccinylation of KIF23 [17]. However, more in‐depth and systematic research needs to be conducted, particularly regarding its role in regulating cell viability, oxidative stress response, and signal transduction pathways, which remain further elucidated.

Emerging evidence indicates that KIF23 may exert its oncogenic functions by activating the Wnt/β‐catenin signaling pathway in various cancers. In chronic myeloid leukemia, KIF23 promotes autophagy‐induced drug resistance by activating Wnt/β‐catenin signaling [18]. Similarly, in esophageal carcinoma, KIF23 overexpression enhances cell proliferation and EMT, whereas its knockdown suppresses these malignant behaviors through inactivation of Wnt/β‐catenin signaling [19]. In nasopharyngeal carcinoma, KIF23 has also been shown to be transcriptionally regulated by the androgen receptor, thereby accelerating tumor progression via activation of the Wnt/β‐catenin pathway [20]. Moreover, aberrant activation of Wnt/β‐catenin signaling has also been implicated in ATC progression, being nearly universal across patient cohorts and shown to drive proliferation, migration, and therapy resistance [21–23]. It is possible that KIF23 may contribute to ATC aggressiveness through a similar mechanism that regulates Wnt/β‐catenin signaling.

Ferroptosis, a recently characterized form of regulated cell death driven by iron‐dependent lipid peroxidation, has been increasingly recognized as a key process in ATC biology [24, 25]. Interestingly, recent evidence has shown that ferroptosis resistance in ATC can be mediated by Wnt/β‐catenin signaling, as stabilization of β‐catenin confers protection against ferroptotic cell death [26]. However, no studies to date have examined whether KIF23, which has been implicated in Wnt/β‐catenin regulation in other cancers, might also be linked to ferroptosis in ATC. In this study, we therefore aimed to perform preliminary investigations of both KIF23/Wnt/β‐catenin and KIF23‐ferroptosis axes separately, with the ultimate goal of laying the groundwork for future studies exploring whether KIF23 may promote ATC progression through Wnt/β‐catenin–mediated ferroptosis. Specifically, the effects of KIF23 on cell viability, oxidative stress response, migration, and invasion capabilities were analyzed in this study by constructing ATC cell models with silenced and overexpressed KIF23 genes. Simultaneously, the interactive relationship between KIF23 and the Wnt/β‐catenin signaling pathway and its role in the process of ferroptosis also explored. The outcomes of this research are not only expected to bring more precise and effective treatment options for ATC patients but also to provide new ideas and insights for studies in the field of cancer biology. The present study is compliant with the TITAN guidelines 2025 [27].

## 2. Methods

### 2.1. Cell Culture

The ATC cells BHT101 were purchased from Shanghai Yaji Biotechnology (product number: YS1427) and cultured in DMEM high‐glucose medium (11965118, Gibco, China) containing 10% fetal bovine serum (FSD500, Excell Bio, China) and 100 U/mL penicillin–streptomycin solution (100X) (C0222, Beyotime, China). The cell culture incubator was maintained at 37°C with 5% CO_2_. When the cells reached 80%–90% confluency, they were passaged using 0.25% trypsin‐EDTA solution (R001100, Gibco, China). The present study only performed experiments in commercially purchased cell line, and therefore no ethical approval was required. The BHT101 ATC cell line was selected because it is widely used as an established in vitro model for studying ATC proliferation and invasion characteristics [28].

### 2.2. Cell Transfection and Stimulation

The transfection experiment used the Lipofectamine 3000 transfection kit (Invitrogen, USA), and then the si‐KIF23 plasmid and overexpressed KIF23 plasmid were synthesized by Sangon Biotech (Shanghai) Co., Ltd. Before transfection, cells were seeded in 6‐well plates at a density of 2 × 10^5^ cells/mL, and the final concentration of si‐RNA or plasmid during transfection was 50 nM. Subsequently, 48 h after transfection, the cells were divided into three main parts to comprehensively investigate the various biological functions of KIF23.

Firstly, to investigate the effect of KIF23 on the activation of the Wnt/β‐catenin pathway and ferroptosis, three experimental groups were set up: an untreated control group (CK group), a group with reduced KIF23 expression by transfecting si‐RNA plasmid targeting KIF23 mRNA (si‐KIF23 group), and a group transfected with nontargeting si‐RNA plasmid as a negative control (NC‐siRNA‐KIF23 group). Additionally, there were groups transfected with an empty vector as a negative control (NC‐vector‐KIF23 group), a group transfected with overexpressed KIF23 plasmid (OE‐KIF23 group), and a group treated with the Wnt/β‐catenin pathway inhibitor nitazoxanide (NTZ, 10 μM) for 24 h on this basis (OE‐KIF23 + NTZ group) [29]. Finally, to reveal the effects of KIF23 on ferroptosis inhibition and cell migration and invasion, we also established an untreated control group (CK group), a group treated with the ferroptosis inducer Erastin 10 μM for 24 h (erastin group) [30], a group transfected with overexpressed KIF23 plasmid (OE‐KIF23 group), and a group simultaneously transfected with overexpressed KIF23 plasmid and treated with erastin for 24 h (OE‐KIF23 + Erastin group).

**FIGURE 1:**
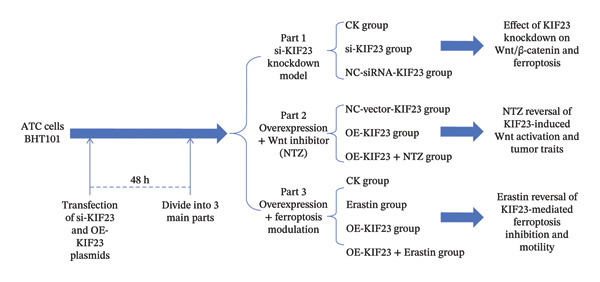
The flowchart of experiments.

NTZ was obtained from Selleckchem (catalog number: S2619), and the erastin was purchased from Sigma‐Aldrich (catalog number: SML0925). In all figures, NC‐KIF23 is used as a general notation for the negative control. In knockdown assays, NC‐KIF23 corresponds to the nontargeting siRNA control (NC‐siRNA), whereas in overexpression assays, it corresponds to the empty vector control (NC‐vector). The flowchart of the experiments is presented in Figure 1.

### 2.3. RT‐qPCR Experiment

Total RNA was extracted using the TRIzol method. Reverse transcription was performed using the HiScript III 1st Strand cDNA Synthesis Kit (R312, Vazyme, China), and qPCR amplification was carried out using Taq Pro Universal SYBR qPCR Master Mix (Q712, Vazyme, China). The reaction conditions were as follows: initial denaturation at 95°C for 30 s, followed by 40 cycles of denaturation at 95°C for 10 s, annealing, and extension at 60°C for 10 seconds. The primer sequences were as follows: KIF23 Forward: 5′‐AGA​GGA​TCA​TTG​CGG​CAG​G‐3′; KIF23 Reverse: 5′‐CTA​GTT​CCT​GGT​GGG​TCA​G‐3′; GAPDH Forward: 5′‐CCC​TTA​AGA​GGG​ATG​CTG​CC‐3′; GAPDH Reverse: 5′‐TAC​GGC​CAA​ATC​CGT​TCA​CA‐3′. All primers were designed based on the *Homo sapiens* reference mRNA sequences for KIF23 and GAPDH and were generated by Sangon Biotech (Shanghai, China). The 2^−ΔΔCt^ method is employed as the computational technique, wherein ΔΔCt signifies the subtraction of the difference in Ct values between the target and reference genes in the control group from the analogous difference observed in the experimental group.

### 2.4. Cell Proliferation Detection Using CCK‐8

Cells were seeded in a 96‐well plate at a density of 2000 cells per well and incubated for 6 h prior to experimental treatment. After 24 h of incubation, 10 μL of CCK8 solution (BA00208, Bioss, China) was added to each well, and the plate was further incubated for 2 h. The OD450 value was then measured using a microplate reader (Multiskan FC, Thermo Scientific).

### 2.5. Detection of Ferroptosis Markers Using ELISA Kits

The levels of cellular glutathione (GSH), malondialdehyde (MDA), and lipid peroxide (LPO) were measured by ELISA kits. Cells in the logarithmic growth phase were seeded in a 6‐well plate at a density of 1 × 10^6^ cells per well. After 24 h of treatment, the cell supernatant was collected, and commercial ELISA kits (Beyotime, China) were used to perform the assay according to the manufacturer’s instructions.

### 2.6. ROS Detection via Flow Cytometry

Cells were seeded evenly into six‐well plates and treated according to the experimental design. When the cells reached approximately 80%–90% confluence, they were washed with PBS and detached with trypsin. Digestion was stopped with complete medium, and the cells were collected and centrifuged at 1200 r/min for 5 min. The cell pellets were resuspended and incubated with the DCFH‐DA working solution prepared in advance. All procedures were performed in the dark. The cells were incubated at 37°C for 20 min and gently inverted every few minutes to ensure adequate probe loading. After incubation, the cells were washed twice with cold PBS to remove excess probe. Finally, the cells were resuspended in PBS and subjected to flow‐cytometric analysis. Fluorescence was detected at an excitation wavelength of 488 nm and an emission wavelength of 525 nm. ROS levels were quantified based on mean fluorescence intensity.

### 2.7. Western Blot

Total cell proteins were extracted using RIPA lysis buffer (P0013B, Beyotime, China), and protein concentrations were determined using the BCA method (Beyotime, China). Equal protein samples were subjected to SDS‐PAGE electrophoresis and transferred to a PVDF membrane (IPVH00010, Sigma‐Aldrich, USA). After blocking with 5% skim milk for 1 h, the membrane was incubated with primary antibodies at 4°C overnight. Following washing with TBST, the membrane was incubated with secondary antibodies at room temperature for 2 h and detected using ECL luminescent liquid (Bio‐Rad, USA). The primary antibodies used included KIF23 (Proteintech, China, Catalog No. 28587‐1‐AP), GPX4 (Proteintech, China, Catalog No. 30388‐1‐AP), phospho‐GSK3β (Ser9) (Cell Signaling Technology, USA, Catalog No. 14630), β‐catenin (Cell Signaling Technology, USA, Catalog No. 37447), c‐Myc (Proteintech, China, Catalog No. 10828‐1‐AP), and GAPDH Rabbit mAb (ABclonal, China, Catalog No. A19056). The Goat Anti‐Rabbit IgG H&L (HRP) (Proteintech, China, Catalog No. SA00001‐4) were used for anti‐KIF23, anti‐GPX4, anti‐c‐Myc, and anti‐GAPDH, and Goat Anti‐Mouse IgG H&L (HRP) (Cell Signaling Technology, USA, Catalog No. 7076) was used for anti‐p‐GSK3β (Ser9) and anti‐β‐catenin. The antibody dilution ratios are shown in Table 1.

**TABLE 1:** Antibody dilution ratios.

Antibodies	Dilution (application)
GSK‐3β	1:1000 (WB)
β‐Catenin	1:1000 (WB)
GPX4	1:1000 (WB)
c‐Myc	1:1000 (WB)
GAPDH	1:10,000 (WB)
Goat anti‐rabbit IgG H&L (HRP)	1:20,000 (WB)
Goat anti‐mouse IgG H&L (HRP)	1:20,000 (WB)

### 2.8. Scratch Assay to Detect Cell Migration Ability

Cells were seeded in a 6‐well plate at 1 × 10^6^ cells/mL density. Once the cells reached 90% confluency, a scratch was made on the cell monolayer using a 200‐μL pipette tip. After washing with PBS, images were taken under a microscope. The cells were then incubated for 24 and 48 h, and images were retaken to calculate the migration distance.

### 2.9. Cell Invasion Ability Detection Using Transwell Assay

Fifty microliters of diluted Matrigel were spread into the Transwell chamber. After solidification, cells were added to the upper chamber at a density of 2 × 10^5^ cells/mL, while the lower chamber was filled with culture medium containing 10% fetal bovine serum. After 24 h of incubation, the cells in the upper chamber were wiped off, and the cells in the lower chamber were stained with crystal violet and photographed under a microscope. The number of invading cells was then counted.

### 2.10. Statistical Analysis

In this study, the data analysis was performed using GraphPad Prism 9 software. All data were expressed as mean ± standard deviation (SD). Normality of data distribution was assessed using the Shapiro–Wilk test, and homogeneity of variance was examined with Levene’s test. Comparisons among multiple groups were conducted using one‐way ANOVA followed by Tukey’s post hoc test. A *p* value less than 0.05 was considered statistically significant. All experiments were performed in triplicate, and each consisting of three technical replicates that were averaged prior to analysis.

## 3. Results

### 3.1. The Impact of KIF23 Gene Silencing on ATC Cell Viability and Oxidative Stress Levels

RT‐qPCR analysis revealed significantly reduced KIF23 mRNA levels in the si‐KIF23‐treated cell group compared to both the untreated CK and NC‐siRNA‐KIF23 groups (Figure 2(a), *p* < 0.001), confirming effective knockdown efficiency. Consistent with this, CCK‐8 assay results demonstrated that cell viability was significantly decreased in the si‐KIF23 group relative to controls (Figure 2(b), *p* < 0.01). Assessment of oxidative stress markers revealed that si‐KIF23 treatment led to a significant reduction in intracellular GSH levels, whereas MDA and LPO levels were significantly elevated compared with controls (Figure 2(c), *p* < 0.001). These changes indicate increased oxidative stress following KIF23 silencing and suggest that KIF23 gene silencing may disturb the balance between intracellular oxidation and antioxidation.

FIGURE 2Effects of KIF23 silencing on ATC cell viability and oxidative stress. (a) RT‐qPCR analysis showed significantly reduced expression of KIF23 mRNA in the si‐KIF23‐treated cell group compared to CK and NC‐siRNA‐KIF23 groups. (b) CCK‐8 assay demonstrated decreased cell viability in the si‐KIF23 group relative to the CK and NC‐siRNA‐KIF23 groups. (c) ELISA test results indicate a decrease in intracellular GSH levels and an increase in MDA and LPO levels in the si‐KIF23 group compared to the NC‐siRNA‐KIF23 group. (d) Measurement of intracellular ROS levels showed a significant increase in the si‐KIF23 group and a decrease in the NC‐siRNA‐KIF23 group. (e) Western blot analysis revealed reduced protein expression levels of GPX4, *p*‐GSK3β, β‐catenin, and c‐Myc in the si‐KIF23 group compared to the CK and NC‐siRNA‐KIF23 groups. ^∗^ *p* < 0.05, ^∗∗^ *p* < 0.01, and ^∗∗∗^ *p* < 0.001; *n* = 3. NC‐KIF23 corresponds to the nontargeting siRNA control (NC‐siRNA).

**Figure:**
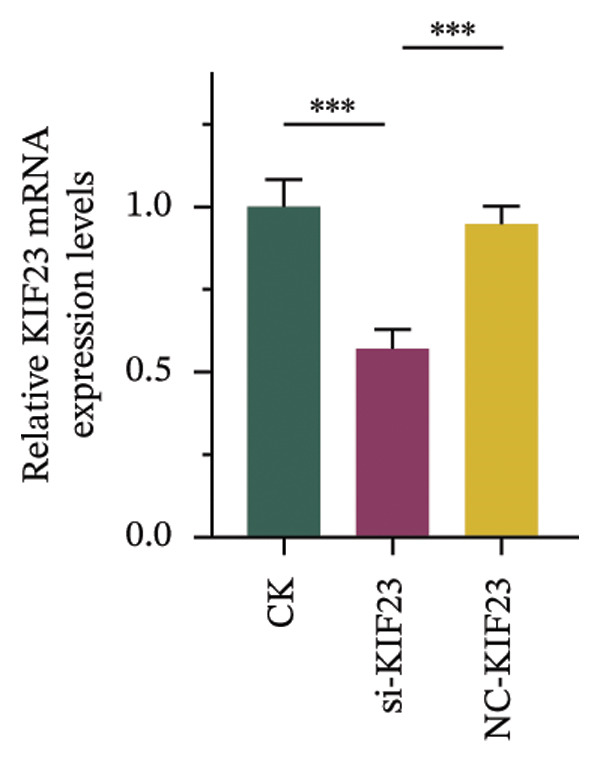
(a)

**Figure:**
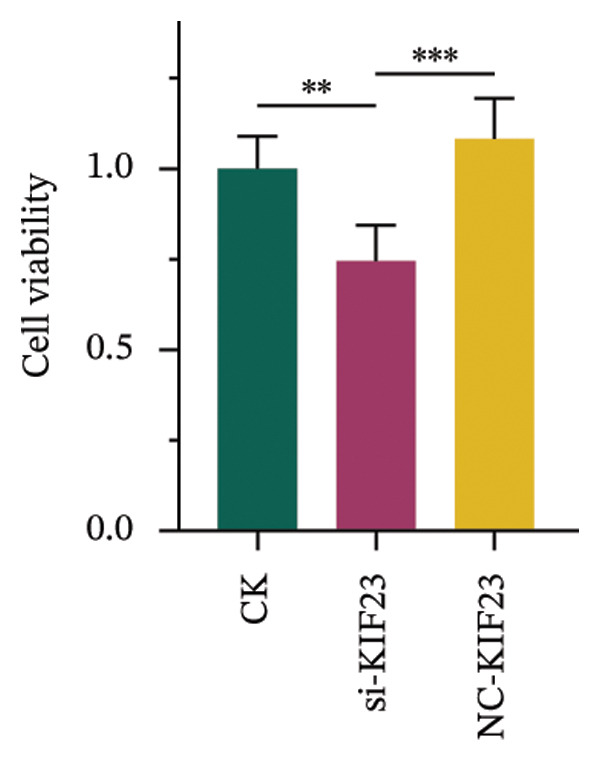
(b)

**Figure:**
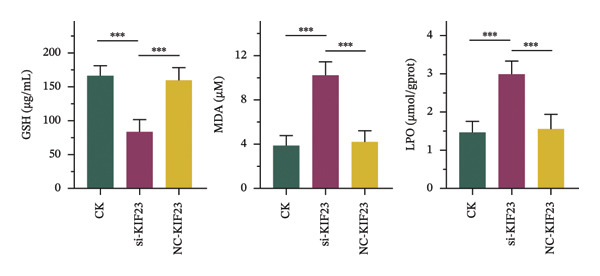
(c)

**Figure:**
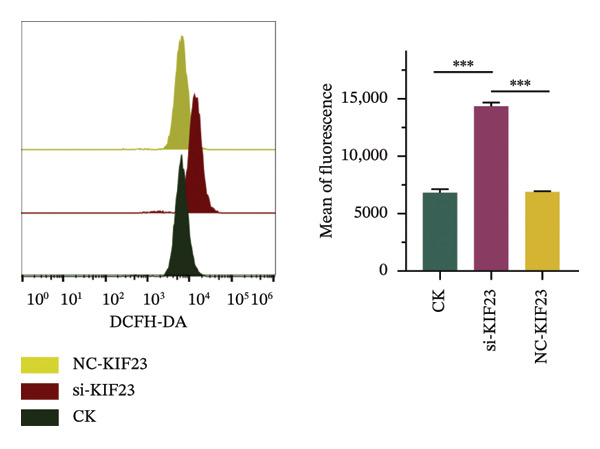
(d)

**Figure:**
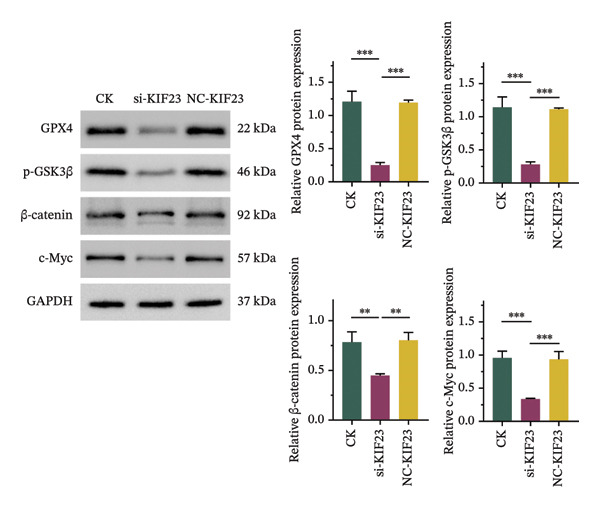
(e)

Flow cytometric analysis further showed that ROS levels were significantly higher in the si‐KIF23 group than in the untreated CK and NC‐siRNA‐KIF23 groups (Figure 2(d), *p* < 0.001). No significant differences were observed between the CK and NC‐siRNA‐KIF23 groups, confirming that the negative‐control treatment did not affect baseline oxidative status. Western blot results showed that the protein expression levels of GPX4, p‐GSK3β, β‐catenin, and c‐Myc were significantly reduced in the si‐KIF23 group compared to the CK group and the NC‐siRNA‐KIF23 group (Figure 2(e), *p* < 0.01). These results suggest that KIF23 may affect the Wnt/β‐catenin signaling pathway and the process of ferroptosis by regulating the expression of these critical proteins.

### 3.2. KIF23 Regulates Invasion and Proliferation of ATC Cells via the Wnt/β‐Catenin Pathway

In order to further explore the regulatory relationship between KIF23 and Wnt/β‐catenin pathway, KIF23 overexpressing cell lines were established. KIF23 mRNA levels were significantly increased in the OE‐KIF23 group compared with both the CK and NC‐vector‐KIF23 groups (Figure 3(a), *p* < 0.01), confirming effective overexpression. No significant difference was observed between the CK and NC‐vector‐KIF23 groups, indicating that the vector control did not influence baseline KIF23 expression (Figure 3(a), *p* > 0.05). KIF23 mRNA levels in the OE‐KIF23 + NTZ group were comparable to the OE‐KIF23 group, suggesting that NTZ treatment did not alter KIF23 transcription (Figure 3(a), *p* > 0.05). Cell viability was significantly higher in the OE‐KIF23 group than in the CK and NC‐vector‐KIF23 groups, whereas OE‐KIF23 + NTZ cells showed decreased viability compared with OE‐KIF23 (Figure 3(b), *p* < 0.001). These results suggest that KIF23 overexpression promotes cell viability, which can be abolished by inhibiting the Wnt/β‐catenin pathway.

FIGURE 3The effects of overexpressing KIF23 on mRNA levels, cell viability, migration, and invasion, and the molecular changes associated with the Wnt/β‐catenin signaling pathway. (a) OE‐KIF23 results in significantly higher mRNA levels than the CK, and the NC‐vector‐KIF23 exhibits a significant decrease in expression. The addition of NTZ further modulates KIF23 mRNA levels in the OE‐KIF23 + NTZ group, as indicated by statistical significance, suggesting that the Wnt/β‐catenin pathway regulates KIF23 expression. (b) The OE‐KIF23 group has significantly enhanced cell viability relative to the CK and NC‐vector‐KIF23 groups, with the OE‐KIF23 + NTZ group showing a significant decrease in cell viability. (c) The OE‐KIF23 group has significantly higher cell migration ability than the CK group, while the NC‐vector‐KIF23 and OE‐KIF23 + NTZ groups show significantly reduced cell migration. (d) The OE‐KIF23 group exhibits significantly higher cell invasion ability compared to the CK and NC‐vector‐KIF23 groups, and the OE‐KIF23 + NTZ group has significantly lower cell invasion ability compared to the OE‐KIF23 group. (e) Western blot analysis indicates that compared to the CK group, the OE‐KIF23 group has significantly increased protein expression levels of p‐GSK3β, β‐catenin, and c‐Myc, while the OE‐KIF23 + NTZ group shows significantly decreased protein expression levels. ^∗^ *p* < 0.05, ^∗∗^ *p* < 0.01, and ^∗∗∗^ *p* < 0.001; *n* = 3. NC‐KIF23 corresponds to the empty vector control (NC‐vector).

**Figure:**
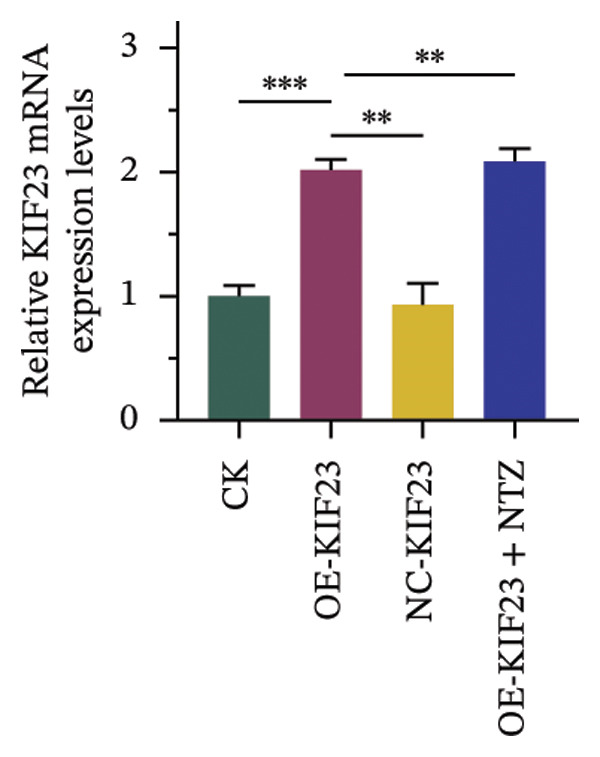
(a)

**Figure:**
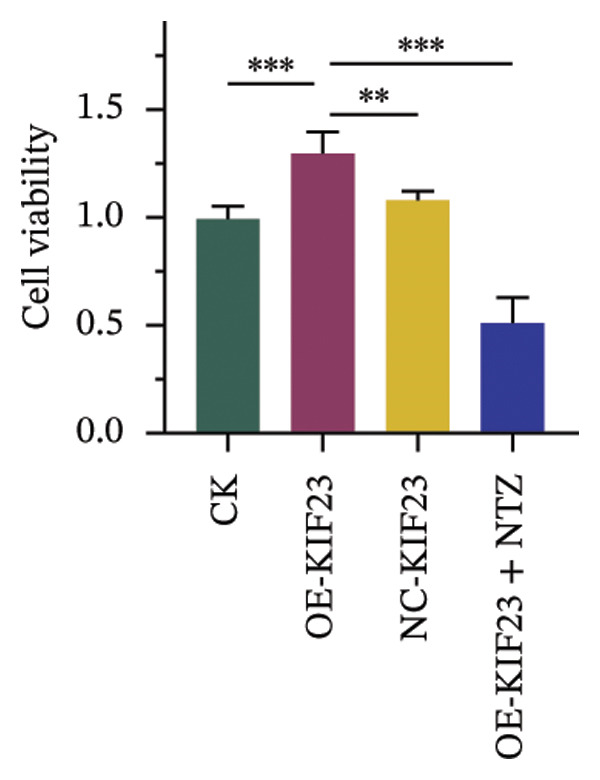
(b)

**Figure:**
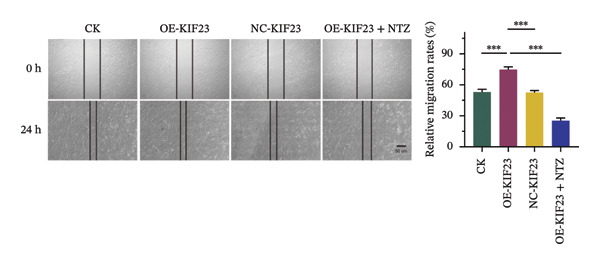
(c)

**Figure:**
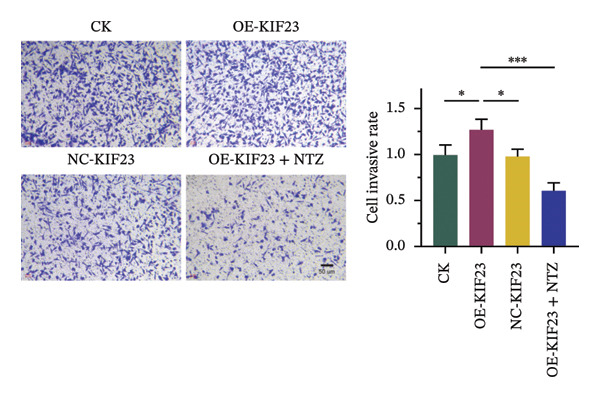
(d)

**Figure:**
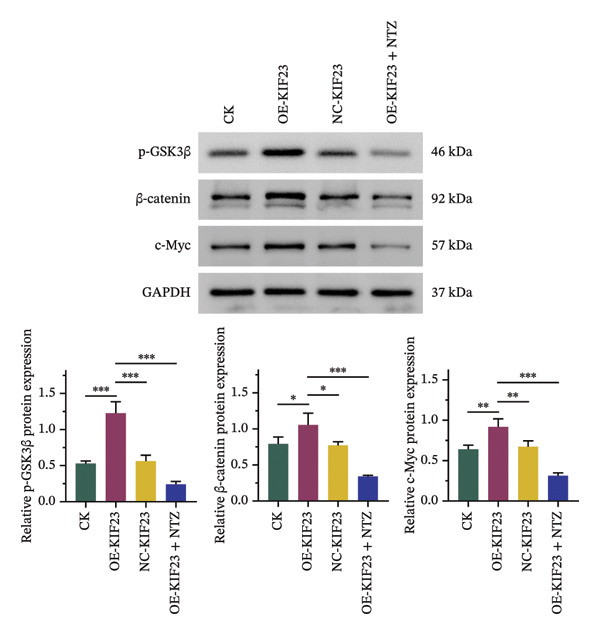
(e)

Scratch assay results demonstrated that the OE‐KIF23 group had significantly higher cell migration ability than the CK group, while OE‐KIF23 + NTZ treatment significantly reduced migration compared with the OE‐KIF23 group (Figure 3(c), both *p* < 0.001). Migration in the NC‐vector‐KIF23 group remained similar to the CK group (Figures 3(c), p*p* > 0.05). Consistent with the cell migration results, Transwell invasion assays revealed that cell invasion ability was markedly increased in the OE‐KIF23 group compared with CK and NC‐vector‐KIF23 (Figure 3(d), *p* < 0.05), whereas OE‐KIF23 + NTZ cells exhibited significantly reduced invasion relative to OE‐KIF23 (Figure 3(d), *p* < 0.001). These results further suggest that KIF23 promotes cell invasion, and the Wnt/β‐catenin pathway also modulates this effect.

Western blot analysis showed that p‐GSK3β, β‐catenin and c‐Myc protein levels were significantly elevated in the OE‐KIF23 group, while NTZ treatment reduced the expression of these proteins compared with OE‐KIF23 (Figure 3(e), *p* < 0.001). No significant changes were observed between the CK and NC‐vector‐KIF23 groups. These findings suggest that KIF23 may affect cell invasion and proliferation by regulating the Wnt/β‐catenin pathway. In summary, KIF23 overexpression significantly enhances cell viability, migration, and invasion abilities, and the Wnt/β‐catenin signaling pathway modulates these effects.

### 3.3. KIF23 Regulates Migration and Invasion of ATC Cells via Ferroptosis Mechanism

RT‐qPCR results showed that KIF23 mRNA expression was significantly increased in the OE‐KIF23 group compared with the CK group (Figure 4(a), *p* < 0.001). No significant difference was observed between CK and erastin‐only groups (Figure 4(a), *p* > 0.05), indicating that erastin did not affect KIF23 transcript levels. Cell viability was significantly higher in the OE‐KIF23 group than in the CK group (Figure 4(b), *p* < 0.01), whereas erastin treatment alone markedly reduced cell viability compared with control (Figure 4(b), *p* < 0.001). Cell viability in the OE‐KIF23 + erastin group was significantly lower than that in OE‐KIF23 (Figure 4(b), *p* < 0.001), indicating that erastin attenuated the pro‐viability effect of KIF23 overexpression.

FIGURE 4The impact of KIF23 overexpression on a range of cellular physiological functions, focusing on its effects in the presence of the ferroptosis inducer, erastin. (a) RT‐qPCR analysis demonstrates a significant increase in mRNA levels in the OE‐KIF23 relative to the CK, with no influence from erastin. (b) Cell viability assays indicate that the OE‐KIF23 group exhibits significantly higher cell viability, which is reduced by erastin treatment (*p* < 0.001). (c) ELISA data show that the OE‐KIF23 group has elevated GSH levels and reduced MDA and LPO levels, effects which are reversed by erastin. (d) ROS content is increased in the OE‐KIF23 group and decreased by erastin treatment. (e) Western blot analysis reveals increased protein expression of p‐GSK3, GPX4, β‐catenin, and c‐Myc in the OE‐KIF23 group, which is reduced by erastin. (f) Scratch assays show increased migration in the OE‐KIF23 group, which is decreased by erastin. (g) Transwell experiments indicate increased invasion in the OE‐KIF23 group, which is decreased by erastin. ^∗^ *p* < 0.05, ^∗∗^ *p* < 0.01, and ^∗∗∗^ *p* < 0.001; *n* = 3.

**Figure:**
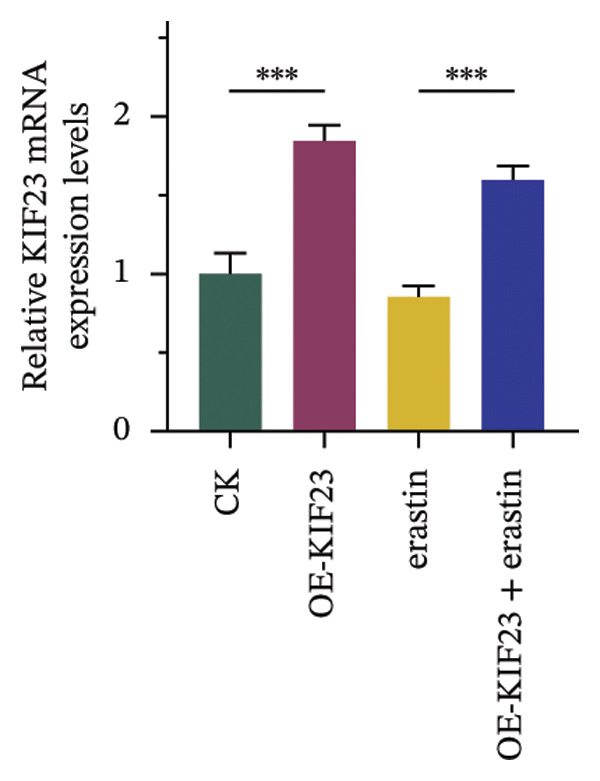
(a)

**Figure:**
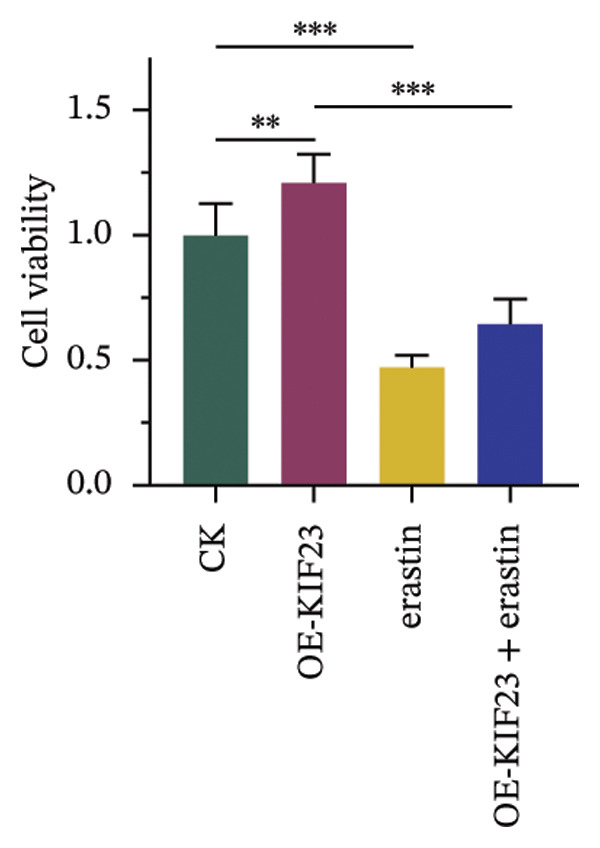
(b)

**Figure:**
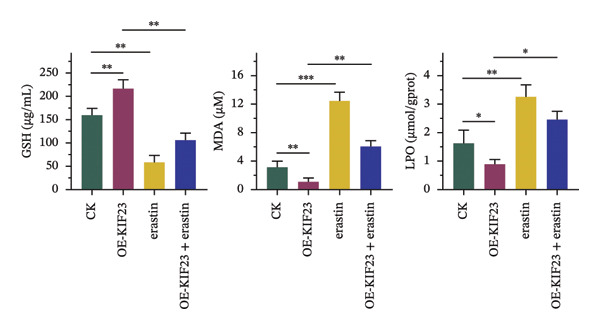
(c)

**Figure:**
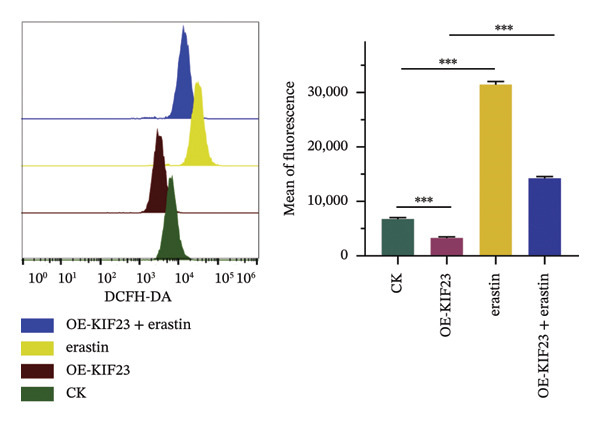
(d)

**Figure:**
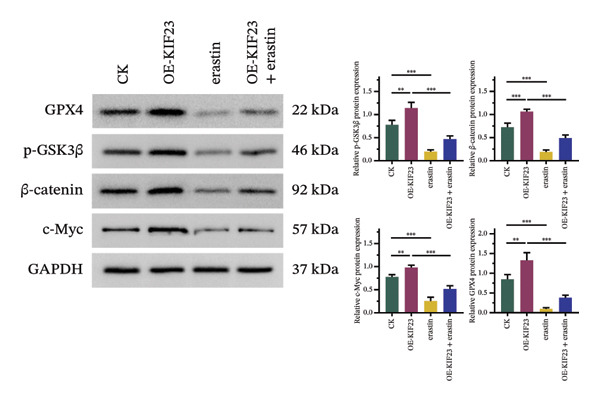
(e)

**Figure:**
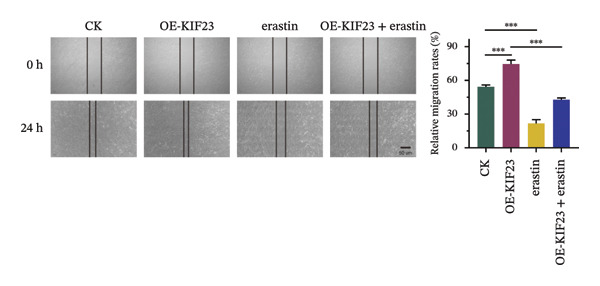
(f)

**Figure:**
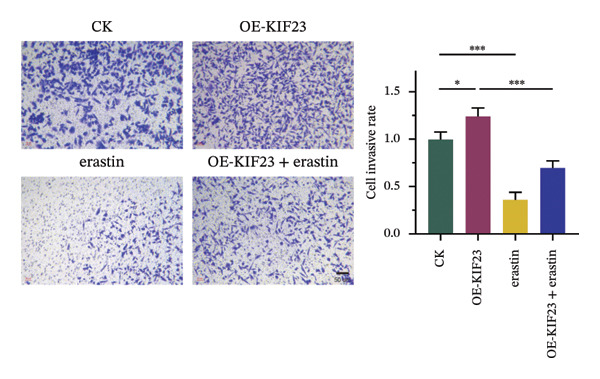
(g)

ELISA results indicated that compared to the CK group, the OE‐KIF23 group had significantly increased GSH levels (Figure 4(c), *p* < 0.01) and significantly decreased MDA and LPO levels (Figure 4(c), *p* < 0.05). In contrast, erastin treatment reduced GSH and increased MDA and LPO levels, and OE‐KIF23 + erastin cells displayed intermediate levels (Figure 4(c), *p* < 0.01), suggesting a partial counteracting effect between KIF23 overexpression and erastin‐induced oxidative stress.

Flow cytometric analysis revealed that compared to the CK group, the OE‐KIF23 group showed a significant decrease in ROS content. In contrast, the erastin‐treated groups had significantly increased ROS levels, and this effect was ameliorated in the OE‐KIF23°+°erastin group (Figure 4(d), *p* < 0.001). This outcome indicates that KIF23 overexpression may inhibit cellular metabolic activities by decreasing ROS production, whereas erastin induces ferroptosis by increasing ROS levels.

Western blot analysis revealed that compared to the CK group, the OE‐KIF23 group had significantly increased protein expression levels of p‐GSK3β, GPX4, β‐catenin, and c‐Myc (Figure 4(e), *p* < 0.01), while the protein expression levels in the erastin‐treated groups were significantly decreased (Figure 4(e), *p* < 0.001). The OE‐KIF23 + erastin group ameliorated the reduction in the expressions of these proteins (Figure 4(e), *p* < 0.001). These results suggest that KIF23 affects cellular physiological functions by regulating the Wnt/β‐catenin pathway and the expression of ferroptosis‐related proteins, and this regulatory effect may be influenced by erastin.

Scratch assays demonstrated that compared to the CK group, the OE‐KIF23 group had significantly increased cell migration ability (Figure 4(f), *p* < 0.001). In contrast, the cell migration ability in the erastin‐treated groups was significantly decreased (Figure 4(f), *p* < 0.001), whereas this effect was ameliorated in the OE‐KIF23 + erastin group. Similar results were obtained from Transwell experiments. Compared to the CK group, the OE‐KIF23 group had significantly increased cell invasion ability (Figure 4(g), *p* < 0.05), while the cell invasion ability in the erastin‐treated groups was significantly decreased (Figure 4(g), *p* < 0.001). Compared to the OE‐KIF23 group, the OE‐KIF23°+°erastin group ameliorated the decrease in cell invasion ability (Figure 4(g), *p* < 0.001). In summary, the overexpression of KIF23 significantly improves cellular physiological functions, and this effect is significantly inhibited under conditions of erastin‐induced ferroptosis.

## 4. Discussion

**FIGURE 5:**
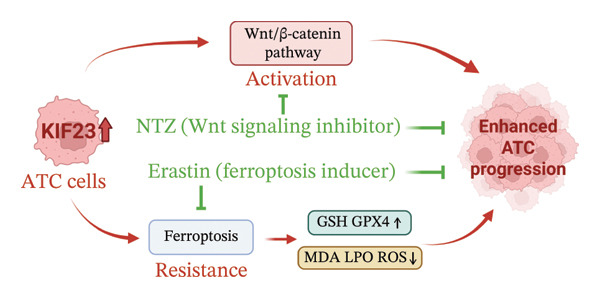
Mechanism of KIF23 in ATC progression. Overexpression of KIF23 promotes ATC cell viability, migration, and invasion. Mechanistically, KIF23 activates the Wnt/β‐catenin signaling pathway, leading to increased nuclear β‐catenin and transcriptional activation of oncogenic targets. KIF23 also contributes to ferroptosis resistance, reflected by elevated GPX4 and reduced lipid peroxidation. Pharmacological interventions with nitazoxanide (NTZ, a Wnt inhibitor) and erastin (a ferroptosis inducer) partially reverse KIF23‐driven phenotypes, suggesting potential therapeutic strategies targeting the KIF23‐Wnt/ferroptosis axis.

ATC is a rare, highly aggressive malignant tumor with a low incidence but a high fatality rate [3]. In this study, we identified KIF23 as a key regulator of ATC cell viability, migration, invasion, and oxidative stress homeostasis. Silencing KIF23 heightened oxidative stress and suppressed malignant properties, whereas KIF23 overexpression enhanced proliferation, motility, and resistance to oxidative injury. Importantly, modulation of KIF23 consistently aligned with changes in Wnt/β‐catenin signaling activity and ferroptosis‐related oxidative responses. The Wnt inhibitor NTZ and the ferroptosis inducer erastin each attenuated the pro‐tumorigenic effects of KIF23 overexpression, supporting the conclusion that KIF23 promotes ATC aggressiveness, at least in part, through coordinated regulation of Wnt signaling and ferroptosis‐associated redox pathways. These discoveries reveal the significant role of KIF23 in tumor cell biology (Figure 5) and suggest that it may emerge as a critical molecular target for future therapeutic strategies.

Previous studies have proven that, as a kinesin family member, the loss of KIF23 expression may lead to disruptions in the internal structure of cells, thereby affecting their survival ability. Saito et al. discovered that KIF23 promotes the proliferation of HCC cells through its interaction with TEAD2 [31]. Yao et al.’s study revealed that high expression of KIF23 is closely associated with T staging and recurrence in bladder cancer patients, and depletion of KIF23 can inhibit the proliferative capacity of bladder cancer cells [32]. Gao et al.’s experimental results demonstrated that knockdown of KIF23 suppresses the proliferation of pancreatic ductal adenocarcinoma cells [33]. In our study, the significant decrease in cell viability observed after specific silencing of the KIF23 gene suggests that KIF23 plays a crucial role in maintaining normal cellular physiological functions, in consistency with these works.

On the other hand, increased KIF23 mRNA levels and enhanced ATC cell viability were observed upon KIF23 overexpression. Recent work by Wu et al. showed that KIF23 promotes ATC cell proliferation and migration through SIRT7‐mediated desuccinylation, but the study did not investigate downstream signaling pathways or cell‐death mechanisms associated with KIF23 activity [17]. Our present findings expand current knowledge by identifying two previously unreported functions of KIF23 in ATC. We demonstrate that KIF23 modulates the Wnt/β‐catenin pathway that is known to be critically involved in ATC aggressiveness and suggest a novel role of KIF23 in ferroptosis regulation. We found that KIF23 overexpression reduces lipid peroxidation and that ferroptosis induction partially reverses KIF23‐driven malignant phenotypes. These results introduce a new conceptual framework in which KIF23 may promote ATC progression through Wnt‐dependent ferroptosis inhibition, thereby extending the current literature beyond the previously described SIRT7‐KIF23 regulatory axis.

Furthermore, the overexpression of KIF23 significantly increases mRNA levels, and this elevation remains unaffected by erastin treatment, suggesting that KIF23 may function through a mechanism independent of the ferroptosis pathway. Ferroptosis, an iron‐dependent programmed cell death, relies on the accumulation of iron ions and the formation of LPOs, leading to excess production of ROS and ultimately triggering cell death [34]. Ferroptosis has demonstrated antitumor effects in various cancers. For instance, METTL17 has been found to influence the sensitivity of colorectal cancer cells to ferroptosis by regulating mitochondrial RNA methylation [35]. The CPT1A/c‐Myc positive feedback loop modulates ferroptosis resistance in lung cancer stem cells via the antioxidant system and lipid metabolism [36]. According to Simona, CD71 involvement in iron internalization enhances the tolerance of ATC cell lines exposed to iron overload toward ferroptosis [37]. The primary mechanisms of ferroptosis include GSH depletion‐based GPX4 inactivation, direct elimination of GPX4, iron ion import, and iron ion reduction. GPX4 is a crucial regulatory factor in ferroptosis, protecting cells from ferroptosis by reducing LPOs. Wang’s research revealed that Vitamin C induces ferritinophagy, leading to the degradation of ferritin and the release of free iron. Excessive iron further triggers the production of ROS through the Fenton reaction, ultimately resulting in ferroptosis of ATC cells [38]. Our findings suggest that KIF23 may maintain the cellular antioxidant capacity by positively regulating the expression of GPX4.

Upon further examination of oxidative stress–related indicators, silencing of the KIF23 gene leads to decreased intracellular GSH levels and increased MDA and LPO levels, which are hallmarks of increased oxidative stress. GSH, a crucial antioxidant, scavenges ROS within cells, protecting them from oxidative damage [38]. Meanwhile, as lipid peroxidation products, MDA and LPO reflect the degree of intracellular oxidative stress [39]. Thus, our findings imply that KIF23 may be involved in regulating the intracellular redox balance. This hypothesis is supported by ROS level detection, where a significant increase in intracellular ROS in the si‐KIF23 group further confirms the intensification of oxidative stress. It is worth noting that the Western blot results revealed a decrease in the expression of GPX4, p‐GSK3β, β‐catenin, and c‐Myc proteins after KIF23 silencing. p‐GSK3β, β‐catenin, and c‐Myc are critical components of the Wnt/β‐catenin signaling pathway, which plays a central role in various physiological processes such as cell proliferation, differentiation, and migration [19, 40, 41]. Ji et al.’s study found that KIF23 activates the Wnt/β‐catenin signaling pathway by promoting the nuclear translocation of β‐catenin, thereby regulating the malignant behavior of colorectal cancer cells [42]. Huang et al.’s research discovered that the silencing of KIF23 inhibits autophagy in imatinib‐resistant chronic myeloid leukemia cells by activating the Wnt/β‐catenin signaling pathway [18]. Xu et al.’s findings indicate that KIF23 can accelerate the progression of nasopharyngeal carcinoma by activating the Wnt/β‐catenin signaling pathway [20].

In summary, this study provides novel evidence that KIF23 promotes ATC progression by modulating Wnt/β‐catenin signaling and ferroptosis. Despite these findings, there are several limitations that should be acknowledged. First, all experiments were conducted *in vitro* with a single ATC cell line and relatively small sample sizes (*n* = 3). Although these assays allowed for preliminary evaluation of KIF23 in regulating cell viability, migration, invasion, oxidative stress, and ferroptosis, the absence of *in vivo* validation limits the translational strength of the conclusions. Future studies incorporating orthotopic or subcutaneous xenograft models and in additional established cell lines (e.g., 8505°C, CAL‐62, and SW‐1736) with larger sample sizes are essential to determine whether KIF23‐driven phenotypes are recapitulated *in vivo* and contribute to tumor growth and metastasis. Second, the mechanistic evidence requires further validation. Direct assays to confirm KIF23‐β‐catenin interactions (e.g., coimmunoprecipitation, TOP/FOP reporter assays, and nuclear fractionation) were not performed. Ferroptosis‐related measurements were limited to LPO and MDA levels, which provide indirect evidence of oxidative stress but do not fully capture dynamic lipid ROS accumulation or pathway specificity. Specific markers such as C11‐BODIPY staining, ACSL4 expression, and iron quantification will be needed, and the paradoxical ROS response to erastin also requires clarification. Third, the potential off‐target effects NTZ on other signaling pathways, such as STAT3, were not investigated in this study. Including systematic evaluation of NTZ’s broader molecular effects are needed to confirm its specificity. Overall, future studies might integrate xenograft and patient‐derived models, expand validation across multiple ATC cell lines, employ more specific ferroptosis assays, and analyze clinical specimens to assess correlations between KIF23 expression, molecular alterations, and patient outcomes, thereby strengthening the translational impact of these findings.

## 5. Conclusion

In summary, this study found that overexpressing KIF23 enhances ATC cell viability, migration, and invasion capabilities, and these effects are positively regulated by the Wnt/β‐catenin signaling pathway. Importantly, treatments with the ferroptosis inducer erastin attenuated the enhanced cell viability caused by KIF23 overexpression and counteracted its promotion of cell migration and invasion abilities. The crucial role of KIF23 in ferroptosis suggests that targeted therapeutic strategies against KIF23 may be combined with ferroptosis inducers to achieve better antitumor effects. Pharmacologic inhibition of KIF23, either directly or in combination with ferroptosis inducers, may represent a novel strategy to overcome treatment resistance in ATC. Although further validation in patient specimens and *in vivo* models is required, these results provide a rationale for translational efforts aiming to integrate KIF23‐targeted interventions into future clinical management of ATC.

## Funding

No funding was received for this manuscript.

## Conflicts of Interest

The authors declare no conflicts of interest.

## Data Availability

The datasets used and/or analyzed during the present study are available from the corresponding author on reasonable request.
